# Ten new species of the spider genus *Althepus* Thorell, 1898 from Southeast Asia (Araneae, Ochyroceratidae)

**DOI:** 10.3897/zookeys.776.24432

**Published:** 2018-07-26

**Authors:** Fengyuan Li, Chang Liu, Shuqiang Li

**Affiliations:** 1 Institute of Zoology, Chinese Academy of Sciences, Beijing 100101, China Institute of Zoology, Chinese Academy of Sciences Beijing China; 2 Southeast Asia Biodiversity Research Institute, Chinese Academy of Sciences, Yezin, Nay Pyi Taw 05282, Myanmar Southeast Asia Biodiversity Research Institute, Chinese Academy of Sciences Nay Pyi Taw Myanmar

**Keywords:** Biodiversity, endemism, Psilodercinae, taxonomy, tropical spiders

## Abstract

Spiders of the genus *Althepus* Thorell, 1898 are found throughout Southeast Asia, notable for their long walking legs. Ten new species are reported in this paper from China, Indonesia, Laos and Myanmar: *A.chengmenensis* Li & Li, **sp. n.** (♂♀), *A.cheni* Li & Li, **sp. n.** (♂♀), *A.gouci* Li & Li, **sp. n.** (♂♀), *A.hongguangi* Li & Li, **sp. n.** (♂♀), *A.phousalao* Li & Li, **sp. n.** (♂♀), *A.qianhuang* Li & Li, **sp. n.** (♂♀), *A.qingyuani* Li & Li, **sp. n.** (♀), *A.sepakuensis* Li & Li, **sp. n.** (♂♀), *A.xuae* Li & Li, **sp. n.** (♂♀) and *A.yizhuang* Li & Li, **sp. n.** (♂♀). These species were found in cave entrances and among tree-buttresses, indicating the spiders have a preference for dark and moist environments. All types are deposited in the Institute of Zoology, Chinese Academy of Sciences in Beijing, China (IZCAS).

## Introduction

The spider family Ochyroceratidae Fage, 1912, contains 20 genera and 216 species (World Spider Catalog 2018). They are small, web-spinning spiders, widely distributed in tropical regions worldwide. Among them, species of the genus *Althepus* Thorell, 1898 build their maze-like, fine horizontal sheet webs 20–50 cm above the ground (Deeleman-Reinhold 1995). Before the current study, the genus *Althepus* contains 33 species, most of them confined to Indo-Burma and the Sunda Shelf Islands (World Spider Catalog 2018). Thorell (1898) described the type species, *A.pictus*, from Myanmar. Brignoli (1973) described two species, one from the Philippines and one from India. Deeleman-Reinhold (1985, 1995) described 13 species from Thailand, Borneo, and Indonesia. Wang and Li (2013) described one species from China. Li et al. (2014) described five species from Laos, Thailand, Myanmar, and Malaysia. Recently, Liu et al. (2017) described eleven species from Thailand.

In this paper, descriptions of ten new *Althepus* species are provided, based on specimens collected from China, Indonesia, Laos, and Myanmar. The genital organs of the males and females are described and images are provided.

## Materials and methods

All spiders are preserved in a 95% ethanol solution. All types are deposited in the Institute of Zoology, Chinese Academy of Sciences in Beijing (IZCAS). Specimens were examined and measured using a Leica M205 C stereomicroscope. Further details were studied with an Olympus BX41 compound microscope. Photos were taken with an Olympus C7070 wide zoom digital camera (7.1 megapixels) mounted on an Olympus SZX12 stereomicroscope. The images were prepared using Helicon Focus 3.0 image stacking software and further processed with Adobe Photoshop. The map was generated in Arcview 3.3. Leg measurements are shown as total length (femur, patella, tibia, metatarsus, tarsus). Leg segments were measured from their retrolateral side. All measurements are given in millimetres (mm). Spider terminology follows that of Li et al. (2014) and Deeleman-Reinhold (1995).

## Taxonomy

### Family Ochyroceratidae Fage, 1912

#### Genus Althepus

Taxon classificationAnimaliaAraneaeOchyroceratidae

Thorell, 1898

Althepus : Thorell 1898: 271–378. Type species Althepuspictus Thorell, 1898 (by original designation), Myanmar.

##### Emended diagnosis.

The genus *Althepus* belongs to the subfamily Psilodercinae and can be distinguished from other genera of Psilodercinae by the following combination of characters: cheliceral promargin with lamina and 1–2 teeth, retromargin with 1–2 small teeth; tarsus of male palp with lateral protrusion bearing a hook-shaped spine; short bulb with embolus; and female internal genitalia often with paired spermathecae (Deeleman-Reinhold 1995).

##### Remarks.

According to our observations, we use hook-shaped spine instead the “lanceolate apophysis” used by Deeleman-Reinhold (1995), and use lamina and 1–2 teeth instead the “3 teeth” used by Deeleman-Reinhold (1995).

#### Althepus chengmenensis

Taxon classificationAnimaliaAraneaeOchyroceratidae

Li & Li sp. n.

http://zoobank.org/67BEA3D7-D377-45D8-8D9F-3A3D582CA643

**Figure 1.**
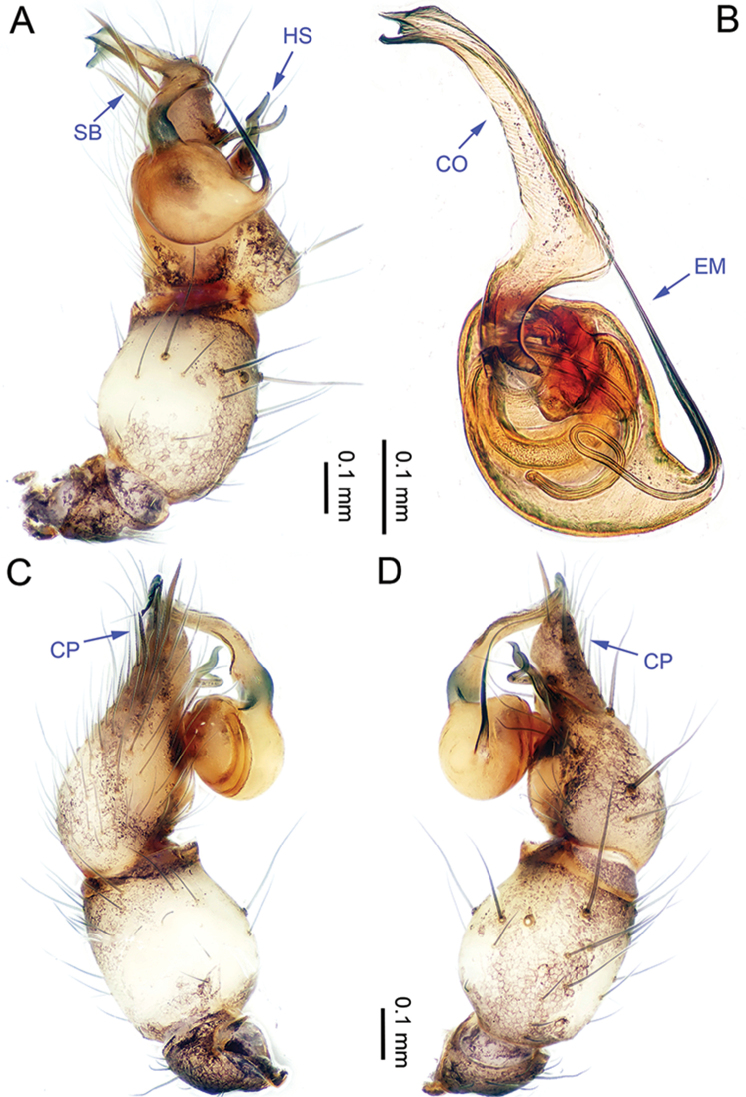
*Althepuschengmenensis* Li & Li, sp. n., male holotype. **A** Palp, ventral view **B** Palpal bulb, ventral view **C** Palp, prolateral view **D** Palp, retrolateral view. Abbreviations: CO conductor; EM embolus; CP cymbial protrusion; HS hook-shaped spine; SB serrated bristles.

**Figure 2.**
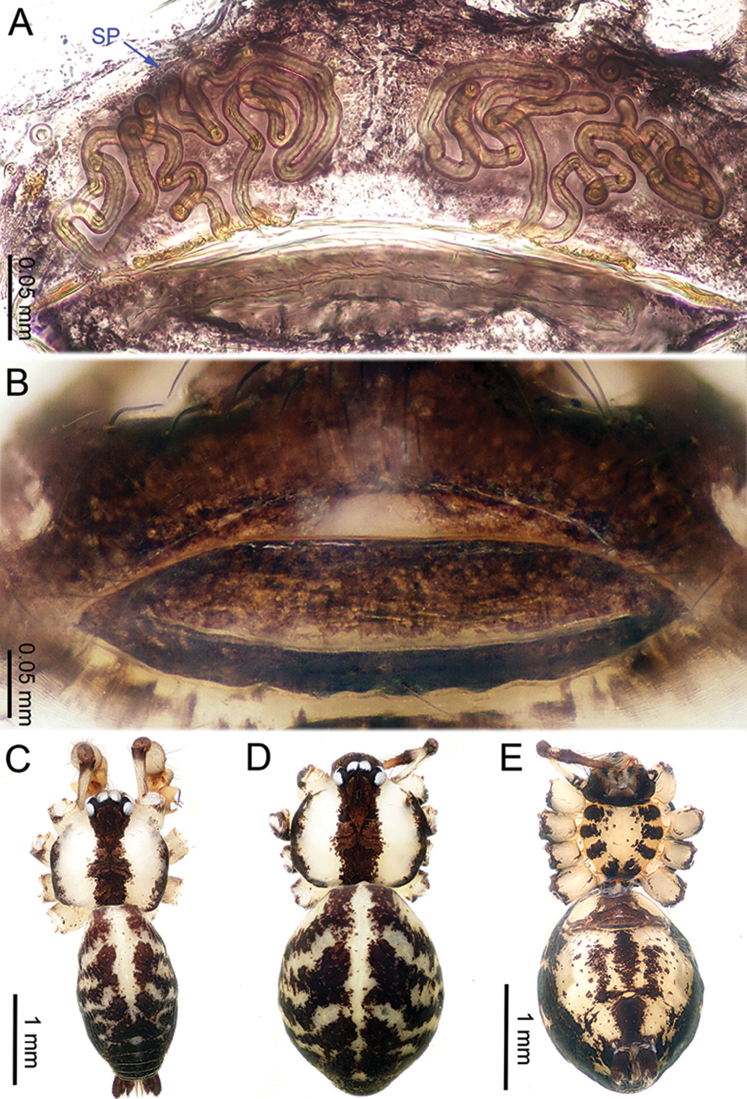
*Althepuschengmenensis* Li & Li, sp. n., male holotype and female paratype. **A** Internal genitalia, dorsal view **B** Female epigastric furrow, ventral view **C** Male habitus, dorsal view **D** Female habitus, dorsal view **E** Female habitus, ventral view. Abbreviation: SP spermatheca.

Figs 1 , 2 , 20 , 21

##### Types.

**Holotype**: ♂, China, Yunnan Province, Baoshan City, Longyang District, Chengmen Cave, 24°55.691'N, 98°45.112'E, 2393 m a.s.l., 14.VII.2016, Y. Li and M. Xu. **Paratypes**: 1♂3♀, same data as holotype.

##### Etymology.

The specific name refers to the type locality; adjective.

##### Diagnosis.

*Althepuschengmenensis* Li & Li, sp. n. resembles *A.xuae* Li & Li, sp. n. in having a sigmoid conductor in males and curved, elongate spermathecae in females. Males can be distinguished from the latter species by the pleated margin of the conductor and by the three acuminate projections on the distal end of the conductor (Figure 1B); females can be distinguished by having longer spermathecae (versus shorter in *A.xuae* Li & Li, sp. n.) (Figure 2A). This new species can be distinguished from all the other known species of the genus by the bent middle part of the conductor (Figure 1A) and by the elongate spermathecae of similar lengths (Figure 2A).

##### Description.

**Male** (holotype). Total length 3.44; carapace 1.14 length, 1.28 width; abdomen 1.95 length, 1.13 width. Carapace round, light yellow, with brown lateral margins and one wide, brown median band, the middle one wider than others (Figure 2C). Anterior margin of cephalic region distinctly elevated. Clypeus brown. Cheliceral promargin with one tooth, followed by a lamina, retromargin with two small teeth (Figure 20A), posterior surface of fang with 25 small denticles. Labium brown. Sternum yellow, with eight brown spots. Abdomen elongate, with complex patterns dorsally and ventrally. Legs brown, femur and tibia with white annulations (Figure 2C). Leg measurements: I 22.56 (5.26, 0.55, 6.15, 8.65, 1.95), II 15.48 (4.23, 0.52, 4.15, 5.10, 1.48), III missing, IV missing. Male palp (Figure 1A–D): tarsus with three slightly curved, serrated bristles at the top of cymbial protrusion (one of them was missing, Figure 1A), one curved spine and one twisted spine with the tip directed towards distally (Figure 1A); bulb yellow, ovate; embolus arising retrolatero-proximally from bulb, slightly sigmoid; conductor arising distally from bulb, sigmoid, with three acuminate projections distally (Figure 1B); embolus and conductor widely separated (distance almost equal to diameter of bulb).

**Female** (one of the paratypes). Total length 3.25; carapace 1.06 length, 1.28 width; abdomen 1.95 length, 1.13 width. Similar to male in colour and general features (Figure 2D–E) but smaller. Internal genitalia with two spermathecae on each side (Figure 2A). Leg measurements: I missing, II 10.82 (3.00, 0.47, 2.75, 3.30, 1.30), III 10.79 (2.95, 0.45, 2.80, 3.40, 1.19), IV 11.42 (3.20, 0.47, 3.15, 3.30, 1.30).

##### Variation.

Males: carapace 1.14–1.38 length, 1.28–1.50 width, femur I 5.26–6.73 (the number of specimens = 2). Females: carapace 1.06–1.20 length, 1.13–1.28 width, femur I 2.88–3.91 (the number of specimens = 3).

##### Distribution.

China. Known only from the type locality (Figure 21).

##### Natural history.

Collected on rocks outside a cave at an altitude of 2393 m.

#### Althepus cheni

Taxon classificationAnimaliaAraneaeOchyroceratidae

Li & Li sp. n.

http://zoobank.org/0A01B8A6-7DFB-4A55-9942-451C5DA62583

**Figure 3.**
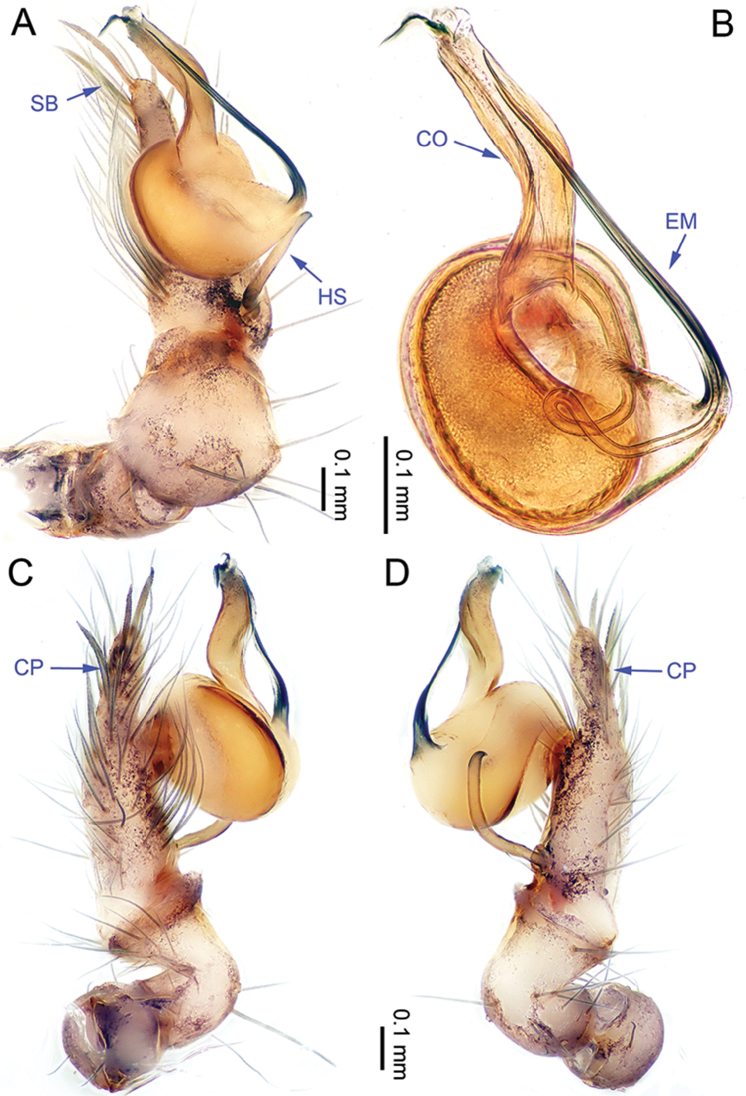
*Althepuscheni* Li & Li, sp. n., male holotype. **A** Palp, ventral view **B** Palpal bulb, ventral view **C** Palp, prolateral view **D** Palp, retrolateral view. Abbreviations: CO conductor; EM embolus; CP cymbial protrusion; HS hook-shaped spine; SB serrated bristles.

**Figure 4.**
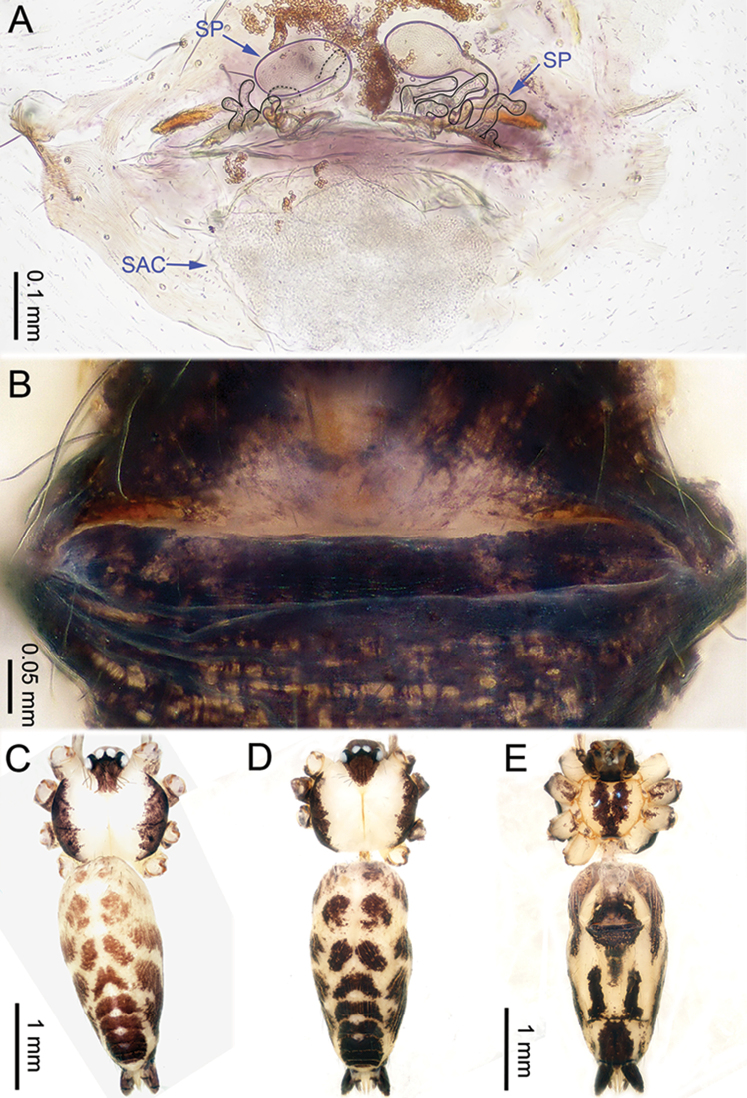
*Althepuscheni* Li & Li, sp. n., male holotype and female paratype. **A** Internal genitalia, dorsal view **B** Female epigastric furrow, ventral view **C** Male habitus, dorsal view **D** Female habitus, dorsal view **E** Female habitus, ventral view. Abbreviation: SP spermatheca.

Figs 3 , 4 , 20 , 21

##### Types.

**Holotype**: ♂, Myanmar, Kadan Island, 12°29.113'N, 98°27.786'E, 3 m a.s.l., 27.X.2017, Z. Chen. **Paratypes**: 1♂3♀, same data as holotype.

##### Etymology.

The specific epithet is a patronym in honour of Zhigang Chen who collected the types; noun (name) in genitive case.

##### Diagnosis.

*Althepuscheni* Li & Li, sp. n. can be distinguished from all other known species of the genus by the large, curved, spine with tip directed distally of the palpal tarsus (Figure 3A) and by the needle-like projection on the distal end of the conductor in males (Figure 3A, B); females can be distinguished by a large membranous sac extending posteriorly and by two types of spermathecae: six short, curved spermathecae, and one globose spermatheca on a long stalk on each side (Figure 4A).

##### Description.

**Male** (holotype). Total length 4.50; carapace 1.36 length, 1.52 width; abdomen 2.75 length, 1.40 width. Carapace round, light yellow, with brown margins and a narrow, brown median line behind ocular area (Figure 4C). Cheliceral promargin with two teeth, followed by a lamina, retromargin with two small teeth (Figure 20B), posterior surface of fang with 25 small denticles. Labium brown. Sternum yellow with a V-shaped pattern in the middle. Abdomen elongate, with complex patterns dorsally and ventrally (Figure 4C). Legs brown, femur and tibia with white annulations. Leg measurements: I 46.53 (11.22, 0.63, 11.54, 19.49, 3.65), II 29.18 (7.76, 0.63, 7.37, 11.54, 1.88), III 18.59 (5.13, 0.60, 4.87, 6.41, 1.58), IV 25.91 (7.63, 0.63, 7.18, 8.72, 1.75). Male palp (Figure 3A–D): tarsus with three slightly curved, serrated bristles at the top of the cymbial protrusion (one of them was missing, Figure 3A) and one large, curved spine with tip directed distally; lateral protrusion small (Figure 3D); bulb yellow, ovate; embolus arising retrolatero-proximally from bulb, slightly sigmoid; conductor arising distally from bulb, slightly sigmoid, with a needle-like projection distally; embolus and conductor widely separated (distance less than diameter of bulb).

**Female** (one of the paratypes). Total length 4.35; carapace 1.25 length, 1.32 width; abdomen 2.50 length, 1.25 width. Similar to male in colour and general features (Figure 4D–E), but smaller. Internal genitalia with six short spermathecae, one globose spermatheca on a long stalk on each side, and a posterior sac (Figure 4A). Leg measurements: I 30.91 (7.37, 0.53, 7.82, 12.63, 2.56), II 19.88 (5.13, 0.52, 5.00, 7.31, 1.92), III 13.15 (3.60, 0.50, 3.25, 4.55, 1.25), IV 18.98 (5.51, 0.52, 5.13, 6.28, 1.54).

##### Variation.

Males: carapace 1.36–1.45 length, 1.50–1.52 width; femur I 11.22 (the number of specimens = 2; leg I lost in one specimen). Females: carapace 1.17–1.25 length, 1.26–1.38 width; femur I 7.37 (the number of specimens = 3).

##### Distribution.

Myanmar. Known only from the type locality (Figure 21).

##### Natural history.

Collected in a lowland evergreen broad-leaved forest at an altitude of 3 m.

#### Althepus gouci

Taxon classificationAnimaliaAraneaeOchyroceratidae

Li & Li sp. n.

http://zoobank.org/831185ED-343E-43FB-9C24-C111CF892DBE

**Figure 5.**
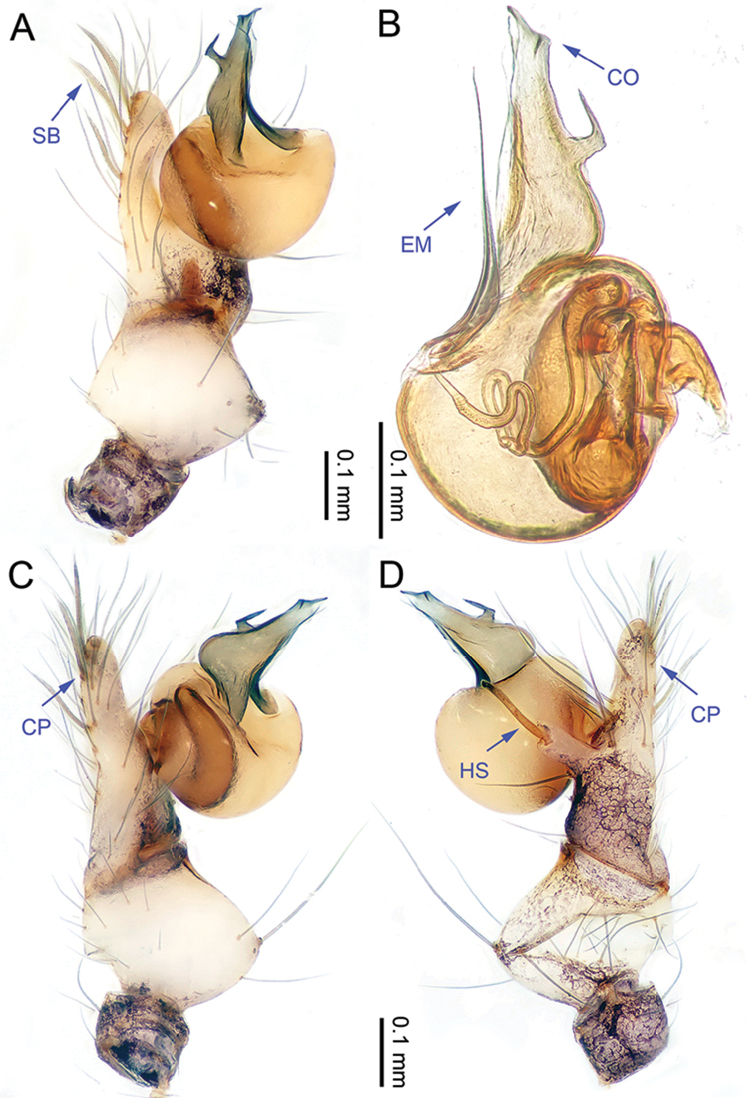
*Althepusgouci* Li & Li, sp. n., male holotype. **A** Palp, ventral view **B** Palpal bulb, retrolateral view **C** Palp, prolateral view **D** Palp, retrolateral view. Abbreviations: CO conductor; EM embolus; CP cymbial protrusion; HS hook-shaped spine; SB serrated bristles.

**Figure 6.**
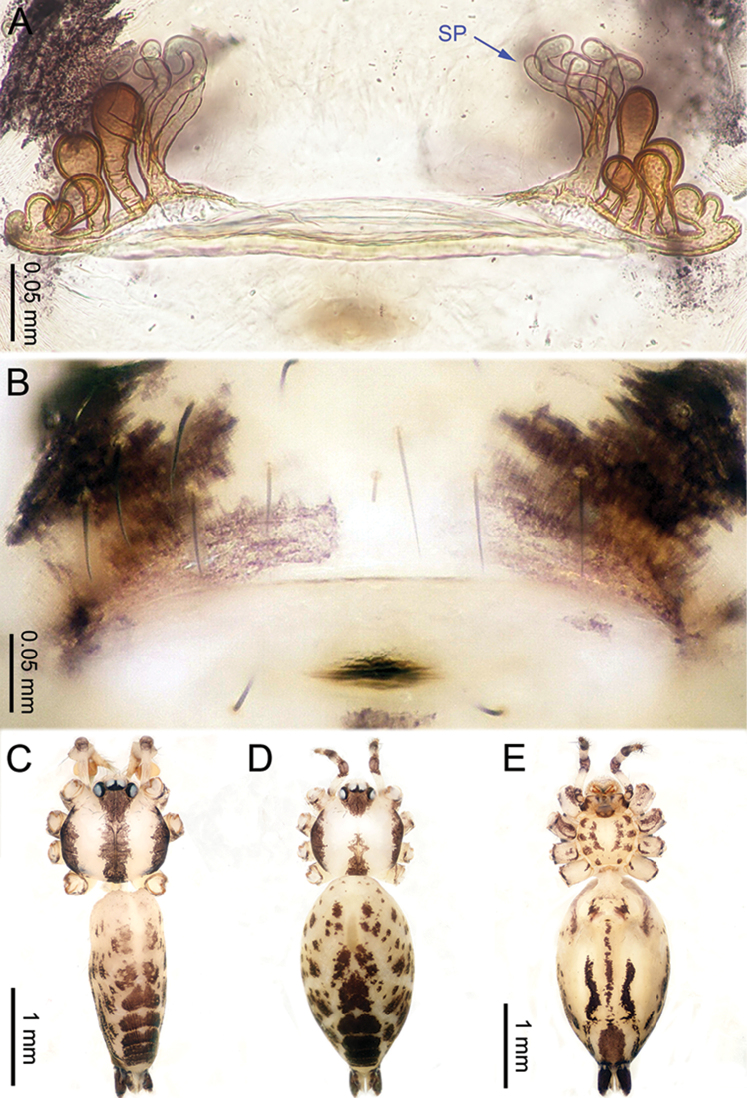
*Althepusgouci* Li & Li, sp. n., male holotype and female paratype. **A** Internal genitali, dorsal view **B** Female epigastric furrow, ventral view **C** Male habitus, dorsal view **D** Female habitus, dorsal view **E** Female habitus, ventral view. Abbreviation: SP spermatheca.

Figs 5 , 6 , 20 , 21

##### Types.

**Holotype**: ♂, Myanmar, Taninthayi Nature Reserve, 14°44.117'N, 98°11.554'E, 307 m a.s.l., 24.X.2017, Z. Chen. **Paratypes**: 2♀, same data as holotype.

##### Etymology.

The specific name is derived from the Chinese pinyin ‘gou ci’, which means ‘hooked spine’, referring to the medially positioned hook-like projection on conductor (Figure 5); noun in apposition.

##### Diagnosis.

*A.gouci* Li & Li, sp. n. can be distinguished from all other known species of the genus by the short embolus and by the hook-like projection on the widened conductor in males (Figure 5); females have two types of spermathecae: one spermatheca with 5–6 curved, long branches, and 5–6 short, thick spermathecae, on each side (two spermathecae with stalks on the left side and four spermathecae with stalks on the right side) (Figure 6A).

##### Description.

**Male** (holotype). Total length 3.44; carapace 1.10 length, 1.15 width; abdomen 1.90 length, 0.88 width. Carapace round, yellow, with brown lateral margins and one wide, brown median band, the middle one wider than the others (Figure 6C). Anterior margin of cephalic region distinctly elevated. Clypeus light-brown. Cheliceral promargin with two teeth, followed by a lamina, retromargin with two small teeth (Figure 20C), posterior surface of fang with 18 small denticles. Labium brown. Sternum yellow, with some small brown spots. Abdomen elongate, with complex patterns dorsally and ventrally (Figure 6C). Legs all missing. Male palp (Figure 5A–D): tarsus with three slightly curved, serrated bristles at the top of the cymbial protrusion (one of them was missing, Figure 5A), and one hooked spine with the tip directed proximally (Figure 5D); bulb light yellow, ovate; embolus arising retrolatero-proximally from bulb, slightly sigmoid, distad; conductor arising distally from bulb, slightly sigmoid, distad, with wide base; embolus and conductor widely separated (distance less than diameter of bulb).

**Female** (one of the paratypes). Total length 3.80; carapace 1.10 length, 1.11 width; abdomen 2.30 length, 1.50 width. Similar to male in colour and general features (Figure 6D, E), but larger. Internal genitalia with two types of spermathecae on each side (Figure 6A). Leg measurements: I 24.61 (5.83, 0.43, 6.09, 10.26, 2.00), II missing, III 10.54 (3.00, 0.40, 2.70, 3.50, 0.94), IV - (4.232, 0.40, 4.17, 5.00, -).

##### Variation.

Females: carapace 0.90–1.10 length, 1.02–1.11 width, femur I 5.83 (the number of specimens = 2; leg I lost in the other specimen).

##### Distribution.

Myanmar. Known only from the type locality (Figure 21).

##### Natural history.

Collected in a tropical evergreen forest at an altitude of 307 m.

#### Althepus hongguangi

Taxon classificationAnimaliaAraneaeOchyroceratidae

Li & Li sp. n.

http://zoobank.org/C60BF344-587E-41B6-918E-34F1E450D22C

**Figure 7.**
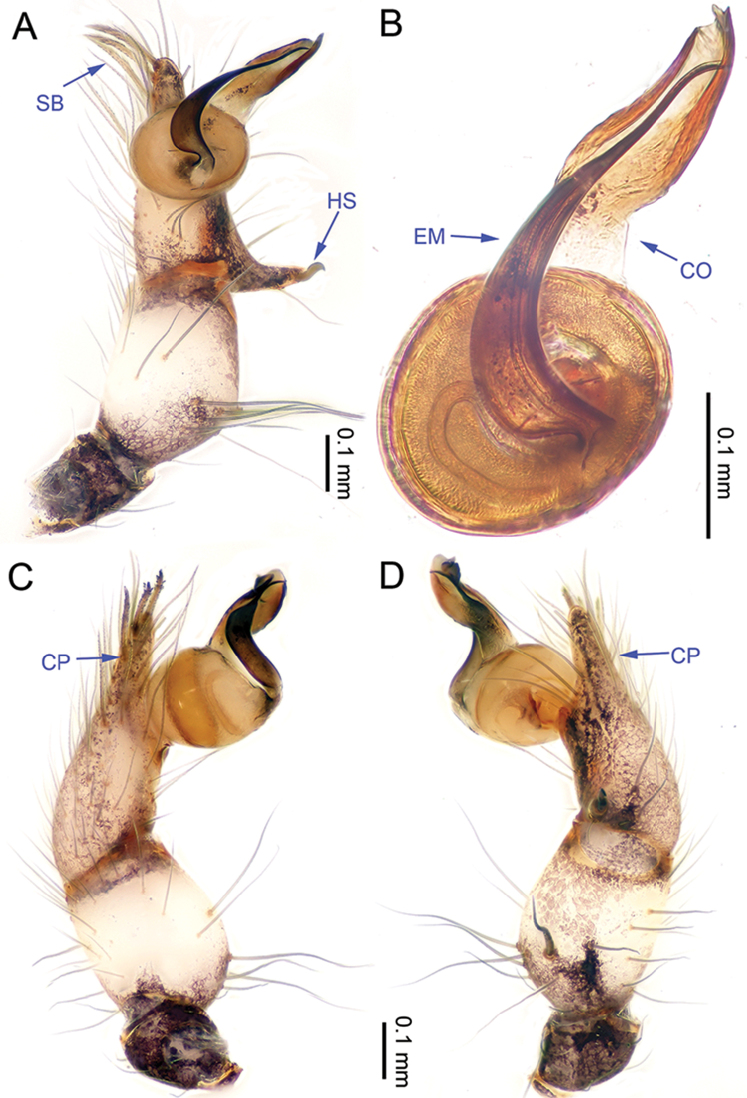
*Althepushongguangi* Li & Li, sp. n., male holotype. **A** Palp, ventral view **B** Palpal bulb, ventral view **C** Palp, prolateral view **D** Palp, retrolateral view. Abbreviations: CO conductor; EM embolus; CP cymbial protrusion; HS hook-shaped spine; SB serrated bristles.

**Figure 8.**
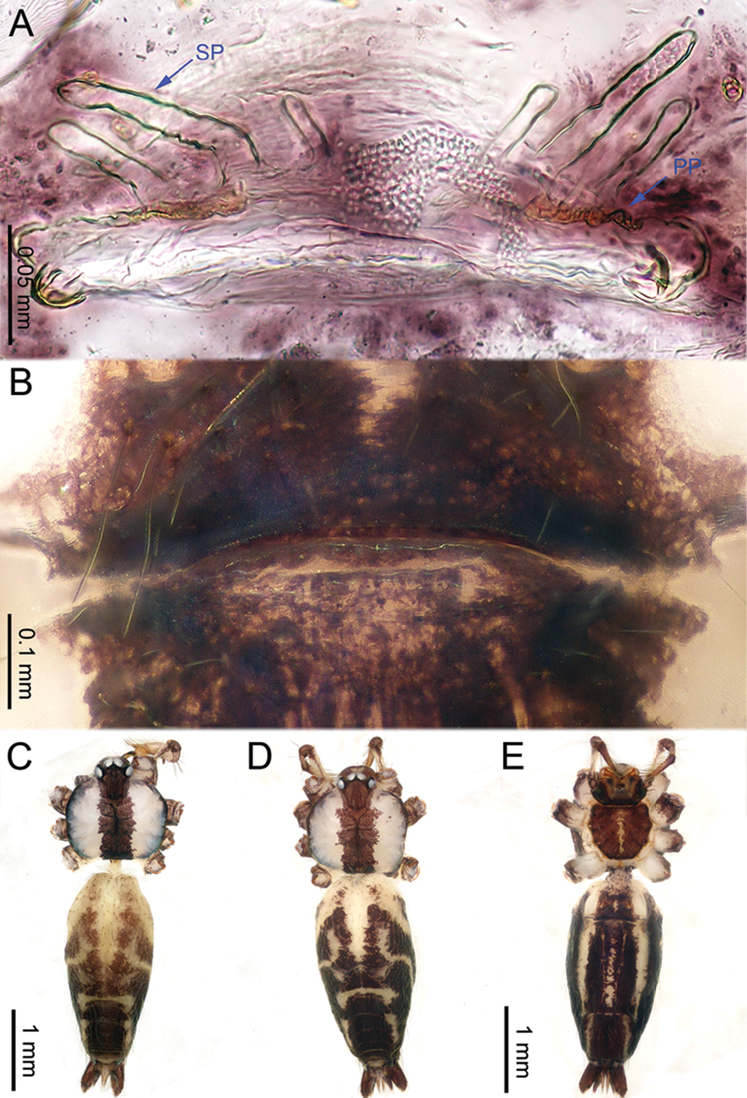
*Althepushongguangi* Li & Li, sp. n., male holotype and female paratype. **A** Internal genitalia, dorsal view **B** Female epigastric furrow, ventral view **C** Male habitus, dorsal view **D** Female habitus, dorsal view **E** Female habitus, ventral view. Abbreviations: SP spermatheca; PP pore plate.

Figs 7 , 8 , 20 , 22

##### Types.

**Holotype**: ♂, Indonesia, Sulawesi, Mountains in Palopo, 02°59.921'S, 120°08.565'E, 465 m a.s.l., 02.IX.2017, H. Liu and Z. Chen. **Paratypes**: 1♂2♀, same data as holotype.

##### Etymology.

The specific epithet is a patronym in honour of Hongguang Liu who collected the types; noun (name) in genitive case.

##### Diagnosis.

*Althepushongguangi* Li & Li, sp. n. can be distinguished from all other known species of the genus by the wider basal area of the embolus in males (Figure 7B); and by three oblique, elongate spermathecae on each side in females (Figure 8A).

##### Description.

**Male** (holotype). Total length 4.20; carapace 1.27 length, 1.27 width; abdomen 2.38 length, 1.15 width. Carapace round, yellow, with narrow, brown lateral margins and one wide, brown median band, the middle one wider than the others (Figure 8C). Anterior margin of cephalic region distinctly elevated. Clypeus brown. Cheliceral promargin with two teeth, followed by a lamina, retromargin with two small teeth, posterior surface of fang with 24 small denticles (Figure 20D). Labium brown. Sternum brown, with a longitudinal yellow band in the middle. Abdomen elongate, with complex patterns dorsally and ventrally (Figure 8C). Legs brown, femur and tibia with white annulations. Leg measurements: I - (11.47, -, -, -, -), II 29.26 (8.00 0.50 7.05, 11.60, 2.11), III missing, IV 24.94 (7.31, 0.51, 6.79, 8.65, 1.68). Male palp (Figure 7A–D): tarsus with three slightly curved, serrated bristles at the top of cymbial protrusion (Figure 7A), one hooked spine with tip directed proximally (Figure 7A); bulb yellow, ovate; embolus arising proximally from bulb, observably sigmoid, distad; conductor arising distally from bulb, oblique, distad; embolus and conductor widely separated (distance almost equal to half diameter of bulb).

**Female** (one of the paratypes). Total length 3.90; carapace 1.16 length, 1.20 width; abdomen 2.25 length, 1.17 width. Similar to male in colour and general features (Figure 8D–E), but smaller. Internal genitalia with three oblique, elongate spermathecae on each side, each side with a pore-plate at its base (Figure 8A). Leg measurements: I 39.41 (9.36, 0.50, 9.10, 16.92, 3.53), II 22.50 (5.77, 0.50, 5.71, 8.72, 1.80), III missing, IV 21.00 (6.02, 0.50, 5.78, 7.05, 1.65).

##### Variation.

Males: carapace 1.25–1.27 length, 1.27–1.30 width, femur I 11.47–12.18 (the number of specimens = 2). Females: carapace 1.16–1.25 length, 1.20–1.41 width, femur I 9.36 (the number of specimens = 2; leg I lost in the other specimen).

##### Distribution.

Indonesia. Known only from the type locality (Figure 22).

##### Natural history.

Collected among tree buttresses at an altitude of 465 m.

#### Althepus phousalao

Taxon classificationAnimaliaAraneaeOchyroceratidae

Li & Li sp. n.

http://zoobank.org/F4E8B6B1-8D7D-427B-BF79-20F8C17C7951

**Figure 9.**
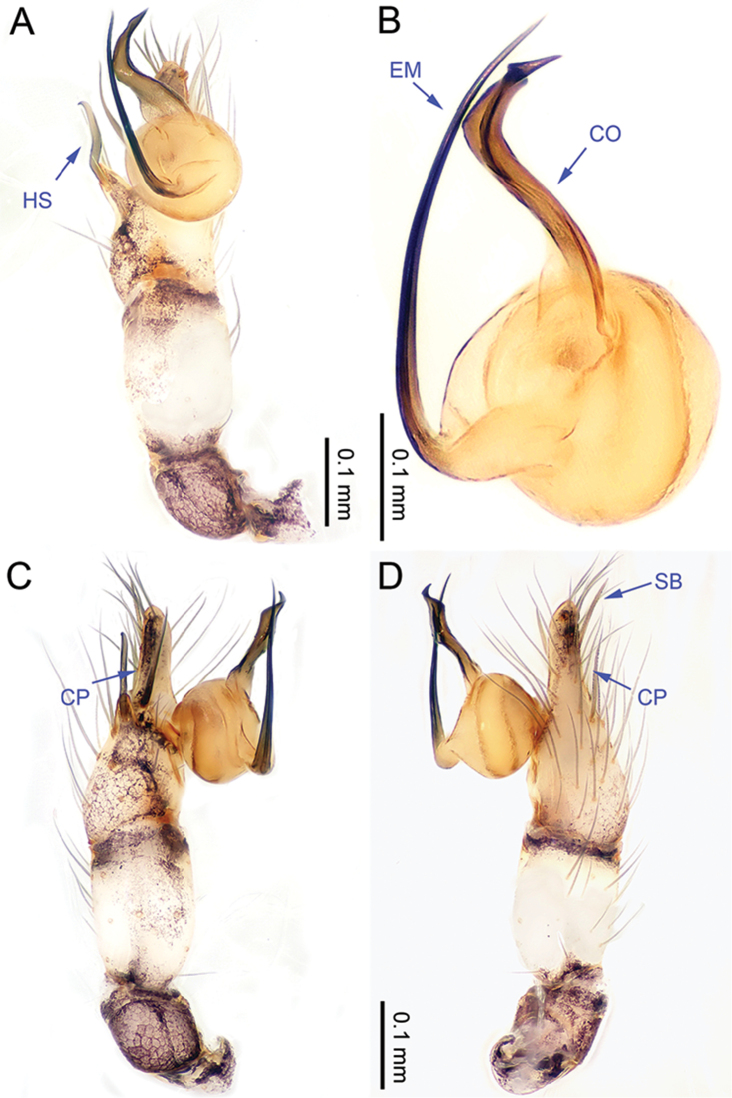
*Althepusphousalao* Li & Li, sp. n., male holotype. **A** Palp, ventral view **B** Palpal bulb, ventral view **C** Palp, retrolateral view **D** Palp, prolateral view. Abbreviations: CO conductor; EM embolus; CP cymbial protrusion; HS hook-shaped spine; SB serrated bristles.

**Figure 10.**
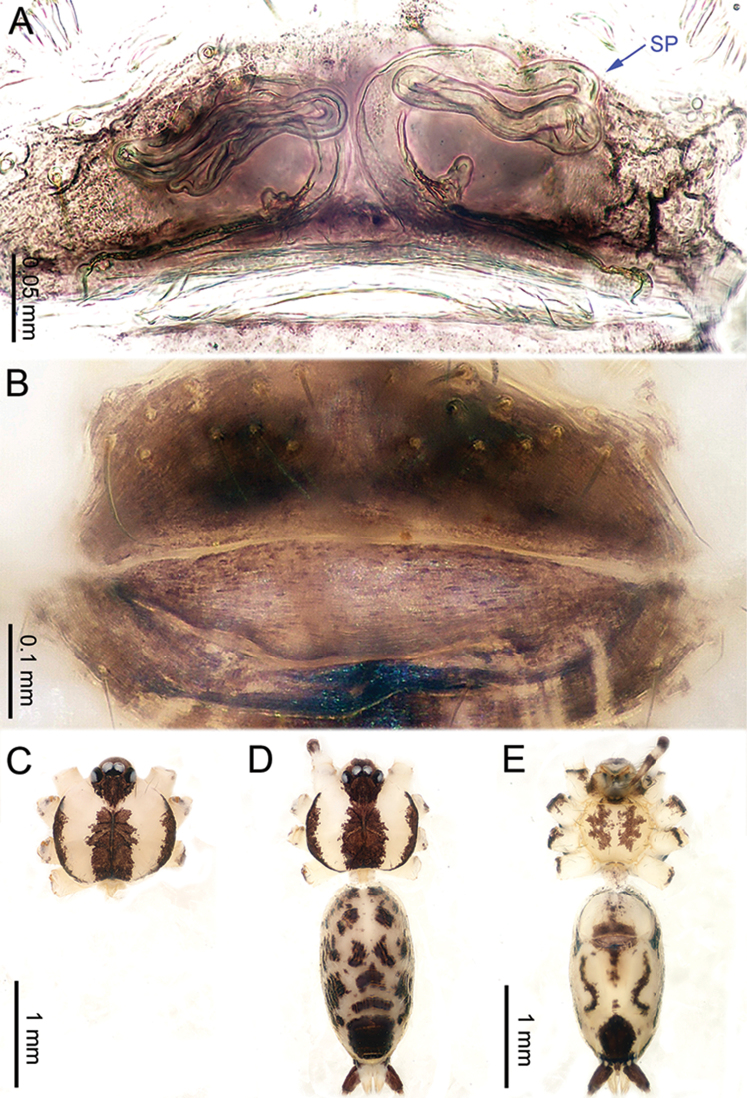
*Althepusphousalao* Li & Li, sp. n., male holotype and female paratype. **A** Internal genitalia, dorsal view **B** Female epigastric furrow, ventral view **C** Male habitus, dorsal view **D** Female habitus, dorsal view **E** Female habitus, ventral view. Abbreviation: SP spermatheca.

Figs 9 , 10 , 20 , 21

##### Types.

**Holotype**: ♂, Laos, Champasak Province, Pakse City, Phou Salao, 15°05.284'N﻿﻿﻿, 105°48.671'E, 242 m a.s.l., 15.XI.2012, Z. Yao. **Paratype**: 1♀, same data as holotype.

##### Etymology.

The specific epithet is a noun in apposition taken from the type locality Phou Salao, Laos.

##### Diagnosis.

*Althepusphousalao* Li & Li, sp. n. resembles *A.leucosternum* Deeleman-Reinhold, 1995, in having a triangular distal end of the conductor and one retrolateral spine of cymbium in males, and one spermatheca on each side in females. Males can be distinguished by the longer conductor (versus shorter in *A.leucosternum*) (Figure 9B). Females can be distinguished by one thicker, longer spermatheca on each side (versus shorter in *A.leucosternum*) (Figure 10A), can be distinguished from all the other known species of the genus by the thick spermathecae (Figure 10A).

##### Description.

**Male** (holotype). Total length -; carapace 1.09 length, 1.17 width; abdomen missing. Carapace round, yellow, with brown lateral margins and one wide, brown median band, the middle one wider than others (Figure 10C). Cheliceral promargin with two teeth, retromargin with two small teeth (Figure 20E), posterior surface of fang with 14 small denticles. Labium light brown. Sternum yellow, with some irregular brown spots. Legs brown, femur and tibia with white annulations. Leg measurements: I missing, II 19.31 (5.45, 0.44, 5.13, 6.79, 1.50), III 13.01 (3.75, 0.44, 3.40, 4.30, 1.12), IV missing. Male palp (Figure 9A–D): tarsus with three slightly curved, serrated bristles at the top of cymbial protrusion (two of them were missing, Figure 9D), one hooked spine with tip directed distally and one long spine retrolaterally (Figure 9A); bulb light yellow, ovate; embolus arising retrolatero-proximally from bulb, slender, slightly curved; conductor arising distally from bulb, observably sigmoid; embolus and conductor widely separated (distance less than diameter of bulb).

**Female** (paratype). Total length 3.20; carapace 0.94 length, 1.13 width; abdomen 1.72 length, 1.00 width. Similar to male in colour and general features of carapace (Figure 10D, E) but smaller. Abdomen elongate, with complex patterns dorsally and ventrally. Internal genitalia with one curved, elongate spermatheca on each side (Figure 10A). Leg measurements: I 26.18 (6.54, 0.48, 7.05, 10.51, 1.60), II missing, III 11.06 (3.25, 0.45, 2.75, 3.55, 1.06), IV 15.84 (4.55, 0.47, 4.36, 5.13, 1.33).

##### Distribution.

Laos. Known only from the type locality (Figure 21).

##### Natural history.

Collected in a pit of Phou Salao at an altitude of 242 m.

##### Remark.

*Althepusphousalao* Li & Li, sp. n., was labelled as “sp. 23” in Li and Li (2018).

#### Althepus qianhuang

Taxon classificationAnimaliaAraneaeOchyroceratidae

Li & Li sp. n.

http://zoobank.org/A20BFBA3-2F97-4A6C-A50C-E618569202A1

**Figure 11.**
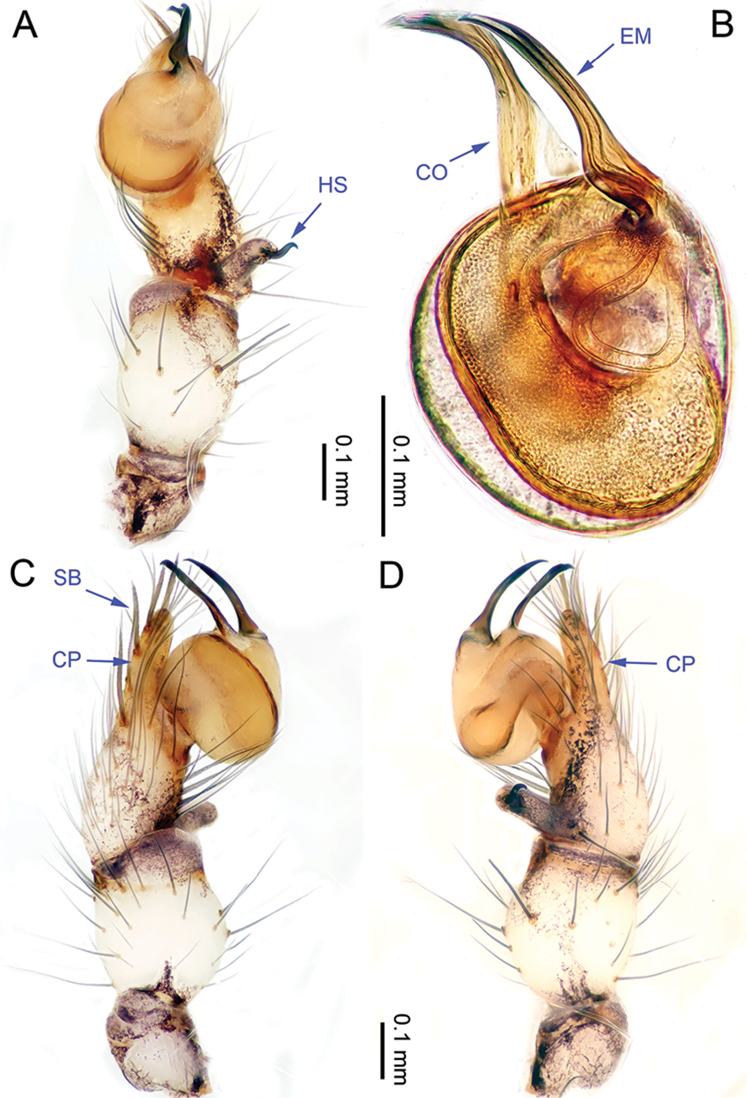
*Althepusqianhuang* Li & Li, sp. n., male holotype. **A** Palp, ventral view **B** Palpal bulb, prolateral view **C** Palp, prolateral view **D** Palp, retrolateral view. Abbreviations: CO conductor; EM embolus; CP cymbial protrusion; HS hook-shaped spine; SB serrated bristles.

**Figure 12.**
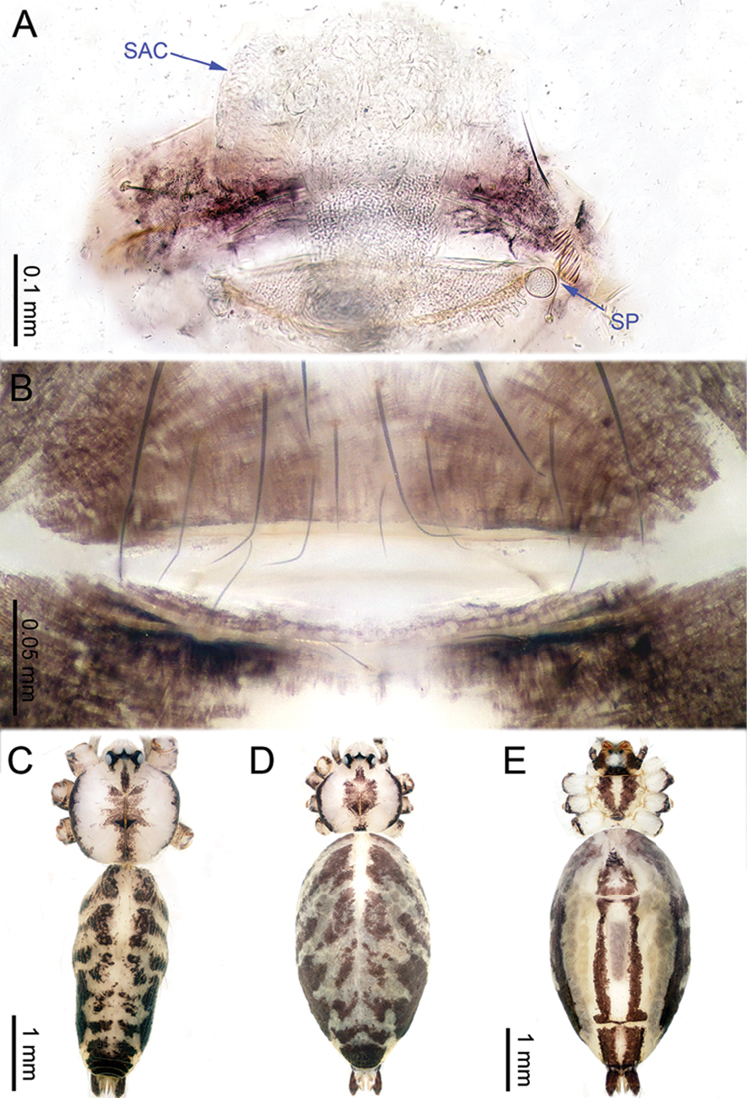
*Althepusqianhuang* Li & Li, sp. n., male holotype and female paratype. **A** Internal genitalia, dorsal view **B** Female epigastric furrow, ventral view **C** Male habitus, dorsal view **D** Female habitus, dorsal view **E** Female habitus, ventral view. Abbreviation: SP spermatheca.

Figs 11 , 12 , 20 , 22

##### Types.

**Holotype**: ♂, Indonesia, Jawa, Special District of Yogyakarta, Kulon Progo Town, Girimulyo, Jatimulyo Village, Gua (Cave) Kiskendo, 7°44.86'S, 110°07.87'E, 662 m a.s.l., 28.VIII.2014, Z. Yao and H. Zhao. **Paratypes**: 2♀, same data as holotype.

##### Etymology.

The specific name is derived from the Chinese pinyin ‘qian huang’, which means ‘pale yellow’, referring to the pale yellow colour of ocular area (Figure 12C, D); adjective.

##### Diagnosis.

*Althepusqianhuang* Li & Li, sp. n. can be distinguished from all other known species of the genus by the nearly parallel conductor and embolus in males (Figure 11); by a large membranous sac extending posteriorly and 1–2 small round spermatheca(e) on each side in the internal genitalia of females (Figure 12A).

##### Description.

**Male** (holotype). Total length 4.49; carapace 1.55 length, 1.48 width; abdomen 2.80 length, 1.31 width. Carapace round, pale yellow, with brown lateral margins and one wide, brown median band, the middle one wider than the others (Figure 12C). Anterior margin of cephalic region distinctly elevated. Cheliceral promargin with two teeth, followed by a lamina, retromargin with two small teeth (Figure 20F), posterior surface of fang with 21 small denticles. Labium brown. Sternum brown. Abdomen elongate, with complex patterns dorsally and ventrally (Figure 12C). Legs brown, femur and tibia with white annulations. Leg measurements: I - (10.13, 0.63, 9.62, 17.31, -), II 25.30 (7.05, 0.63, 6.41, 9.75, 1.46), III 16.19 (4.65, 0.60, 3.92, 5.71, 1.31), IV missing. Male palp (Figure 11A–D): tarsus with one hooked spine with tip directed distally (Figure 11A); bristles at the top of the cymbial protrusion (Figure 11C) as in *A.hongguangi* Li & Li, sp. n.; bulb yellow, ovate; embolus arising distally from bulb, short, slightly curved; conductor arising distally from bulb, short, slightly curved; embolus and conductor slightly separated (distance less than diameter of bulb).

**Female** (one of the paratypes). Total length 4.87; carapace 1.39 length, 1.44 width; abdomen 2.96 length, 1.70 width. Similar to male in colour and general features (Figure 12D–E) but larger. Internal genitalia with 1–2 small round spermatheca(e) on each side and a large posterior sac (Figure 12A). Leg measurements: I missing, II missing, III 12.81 (3.68, 0.46, 3.28, 4.30, 1.09), IV missing.

##### Variation.

Females: carapace 1.17–1.39 length, 1.27–1.44 width; leg I lost (the number of specimens = 2).

##### Distribution.

Indonesia. Known only from the type locality (Figure 22).

##### Natural history.

Collected at a cave entrance at an altitude of 662 m.

##### Remark.

*Althepusqianhuang* Li & Li, sp. n., was labelled as “sp. 119” in the analysis of Li and Li (2018).

#### Althepus qingyuani

Taxon classificationAnimaliaAraneaeOchyroceratidae

Li & Li sp. n.

http://zoobank.org/5967A80C-65AC-46E1-81D0-AC1D1B50BBD0

Figs 13 , 20 , 21

##### Types.

**Holotype**: ♀, China, Yunnan Province, Lincang City, Yongde County, Xiaomengtong Village, Xiangquan Dam, Xianren Cave, 24°12.099'N, 99°18.607'E, 1499 m a.s.l., 02.VIII.2010, C. Wang, L. Lin and Q. Zhao. **Paratype**: 1♀, same data as holotype.

##### Etymology.

The specific name is a patronym in honour of Dr. Qingyuan Zhao who collected the types; noun (name) in genitive case.

##### Diagnosis.

**Figure 13.**
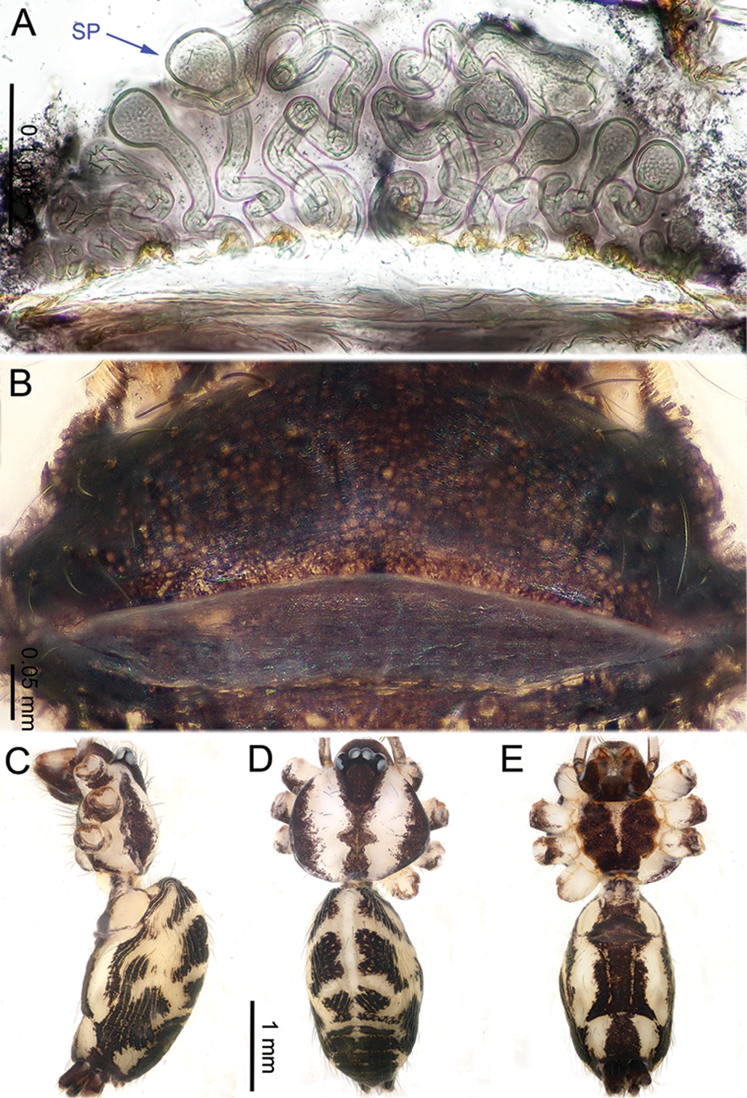
*Althepusqingyuani* Li & Li, sp. n., female holotype. **A** Internal genitalia, dorsal view **B** Female epigastric furrow, ventral view **C** Female habitus, retrolateral view **D** Female habitus, dorsal view **E** Female habitus, ventral view. Abbreviation: SP spermatheca.

*Althepusqingyuani* Li & Li, sp. n. can be distinguished from all other known species of the genus by 16 round spermathecae on curved stalks in the females (Figure 13A).

##### Description.

**Female** (holotype). Total length 3.95; carapace 1.44 length, 1.58 width; abdomen 2.28 length, 1.33 width. Carapace round, yellow, with three longitudinal brown bands of similar widths (Figure 13D–E). Anterior margin of cephalic region distinctly elevated. Clypeus brown. Cheliceral promargin with two teeth, followed by a lamina, retromargin with two small teeth (Figure 20G), posterior surface of fang with 23 small denticles. Labium brown. Sternum brown with a longitudinal yellow band. Abdomen elongate, with complex patterns dorsally and ventrally (Figure 13D, E). Legs brown, femur and tibia with white annulations. Leg measurements: I missing, II 20.80 (5.67, 0.56, 5.26, 7.63, 1.78), III 14.11 (4.17, 0.56, 3.60, 4.45, 1.33), IV missing. Internal genitalia with 16 round spermathecae on curved stalks (Figure 13A).

**Male.** Unknown.

##### Variation.

Females: carapace 1.17–1.44 length, 1.30–1.58 width; leg I lost (the number of specimens = 2).

##### Distribution.

China. Known only from the type locality (Figure 21).

##### Natural history.

Collected at a cave entrance at an altitude of 1499 m.

##### Remark.

*Althepusqingyuani* Li & Li, sp. n., was labelled as “sp. 97” in the analysis of Li and Li (2018).

#### Althepus sepakuensis

Taxon classificationAnimaliaAraneaeOchyroceratidae

Li & Li sp. n.

http://zoobank.org/598BEAF8-7E62-44FE-99F6-3479BFDAEE5F

**Figure 14.**
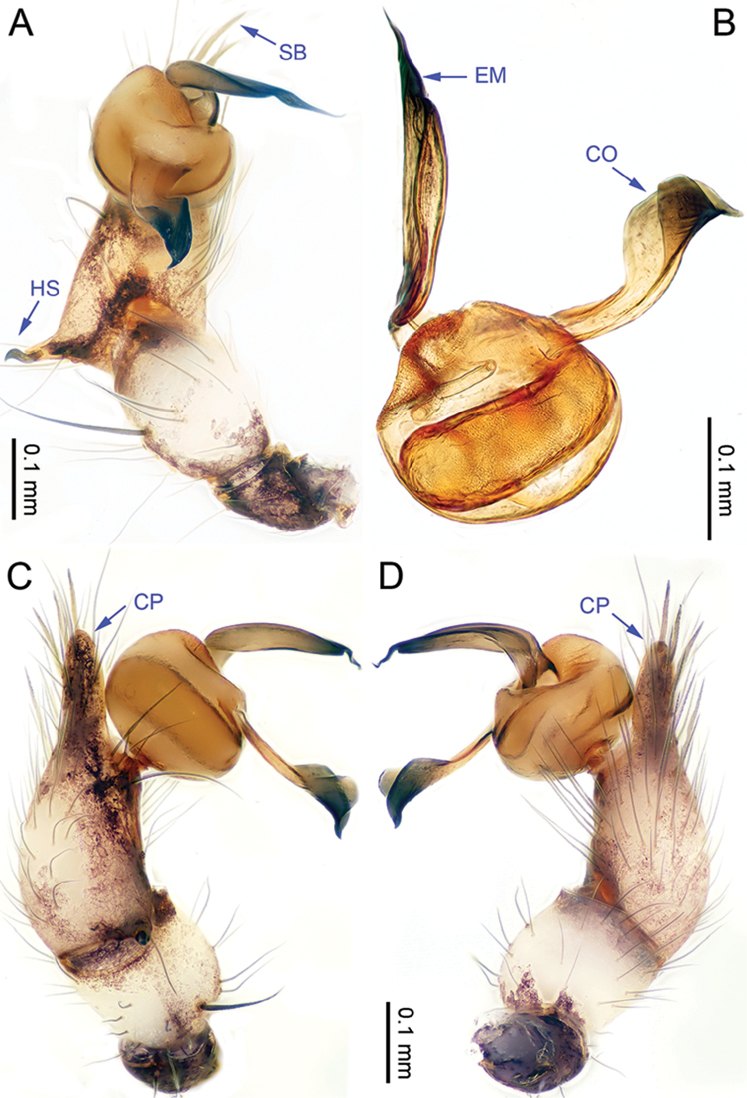
*Althepussepakuensis* Li & Li, sp. n., male holotype. **A** Palp, ventral view **B** Palpal bulb, retrolateral view **C** Palp, retrolateral view **D** Palp, prolateral view. Abbreviations: CO conductor; EM embolus; CP cymbial protrusion; HS hook-shaped spine; SB serrated bristles.

**Figure 15.**
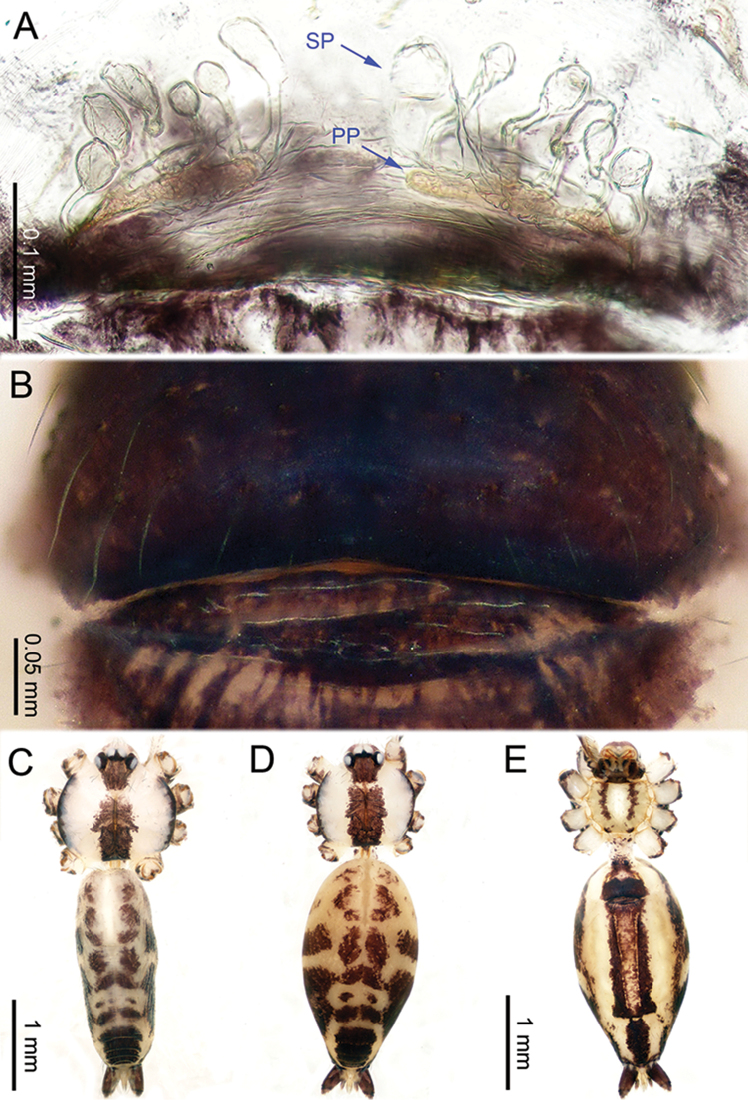
*Althepussepakuensis* Li & Li, sp. n., male holotype and female paratype. **A** Internal genitalia, dorsal view **B** Female epigastric furrow, ventral view **C** Male habitus, dorsal view **D** Female habitus, dorsal view **E** Female habitus, ventral view. Abbreviations: SP spermatheca; PP pore plate.

Figs 14 , 15 , 20 , 22

##### Types.

**Holotype**: ♂, Indonesia, East Kalimantan, Penajam, Paser Utara Town, Sepaku Village, on foot of Gunung Parung, 00°50.920'S, 116°46.284'E, 60 m a.s.l., 17.VIII.2014, H. Zhao and Z. Yao. **Paratype**: 1♀, same data as holotype.

##### Other material examined.

1♂, Indonesia, East Kalimantan, Penajam, Camp of International Timber Corporation of Indonesia, 01°05.291'S, 116°41.009'E, 64 m a.s.l., 17.VIII.2014, H. Zhao and Z. Yao.

##### Etymology.

The specific name refers to the type locality; adjective.

##### Diagnosis.

Males of *A.sepakuensis* Li & Li, sp. n. can be easily distinguished from all other known species of the genus by the widened, laminar embolus with a distal acuminate end (Figure 14B); females, by the six round spermathecae on slender stalks on each side (Figure 15A).

##### Description.

**Male** (holotype). Total length 4.23; carapace 1.33 length, 1.34 width; abdomen 2.25 length, 1.23 width. Carapace round, light yellow, with narrow, brown lateral margins and one wide, brown median band, the middle one wider than the others (Figure 15C). Anterior margin of cephalic region distinctly elevated. Clypeus brown. Cheliceral promargin with two teeth, followed by a lamina, retromargin with two small teeth (Figure 20H), posterior surface of fang with 19 small denticles. Labium brown. Sternum yellow, with two longitudinal brown bands. Abdomen elongate, with complex patterns dorsally and ventrally (Figure 15C). Legs brown, femur and tibia with white annulations. Leg measurements: I 37.7 (9.23, 0.53, 9.29, 15.77, 2.88), II 24.37 (6.35, 0.53, 6.03, 9.62, 1.84), III missing, IV 22.26 (6.41, 0.52, 5.90, 7.88, 1.55). Male palp (Figure 14A–D): tarsus with one hooked spine with tip directed distally (Figure 14A); bristles at the top of cymbial protrusion (Figure 14A) as *A.hongguangi* Li & Li, sp. n.; bulb yellow, ovate; embolus arising distally from bulb, wide, curved, distal part abruptly acute to acuminate; conductor arising proximally from bulb, wide, distal part incurved; embolus and conductor widely separated (distance less than diameter of bulb).

**Female** (paratype). Total length 4.00; carapace 1.05 length, 1.09 width; abdomen 2.34 length, 1.32 width. Similar to male in colour and general features (Figure 15D, E) but smaller. Six rounded spermathecae on slender stalks, adjoined to a large pore-plate on each side (Figure 15E). Leg measurements: I 28.24 (6.54, 0.43, 6.72, 11.67, 2.88), II 17.45 (4.49, 0.43, 4.30, 6.60, 1.63), III 11.58 (3.36, 0.40, 2.81, 3.88, 1.13), IV 16.31 (4.55, 0.43, 4.25, 5.64, 1.44).

##### Distribution.

Indonesia. East Kalimantan, Penajam (Figure 22).

##### Natural history.

Collected in a lowland tropical forest.

##### Remark.

*Althepussepakuensis* Li & Li, sp. n., was labelled as “sp. 131” in the analysis of Li and Li (2018).

#### Althepus xuae

Taxon classificationAnimaliaAraneaeOchyroceratidae

Li & Li sp. n.

http://zoobank.org/E0535C6D-7C71-4AFB-9197-7A9851B4B145

**Figure 16.**
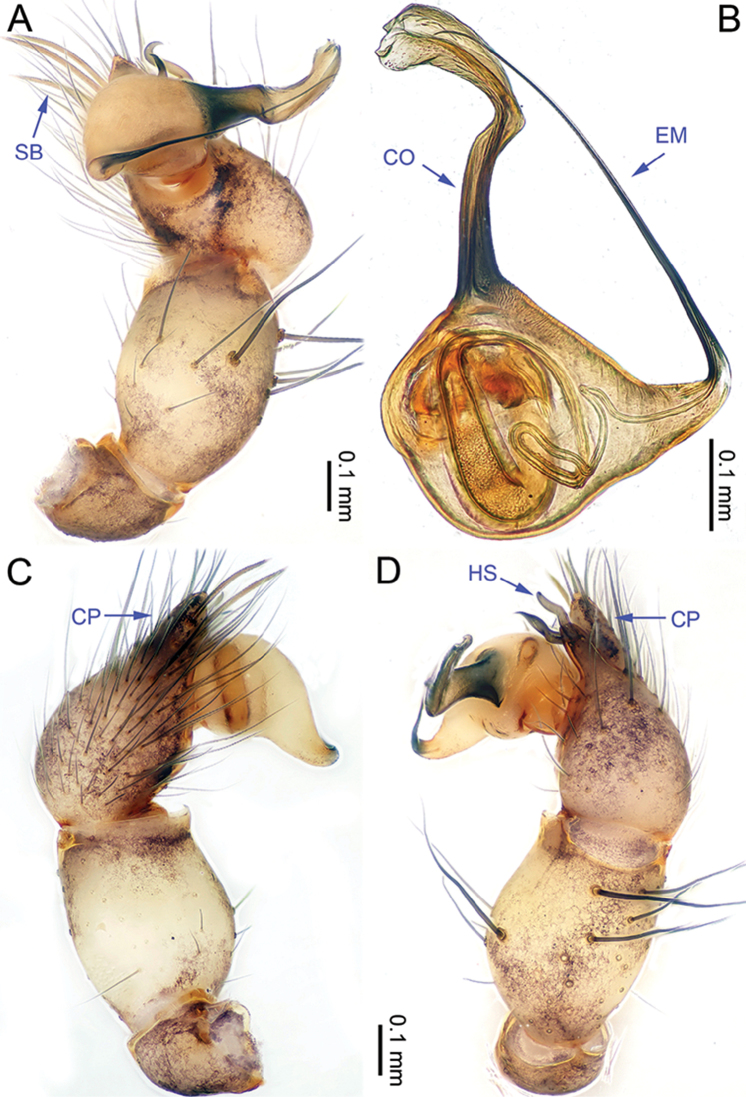
*Althepusxuae* Li & Li, sp. n., male holotype. **A** Palp, ventral view **B** Palpal bulb, prolateral view **C** Palp, prolateral view **D** Palp, retrolateral view. Abbreviations: CO conductor; EM embolus; CP cymbial protrusion; HS hook-shaped spine; SB serrated bristles.

**Figure 17.**
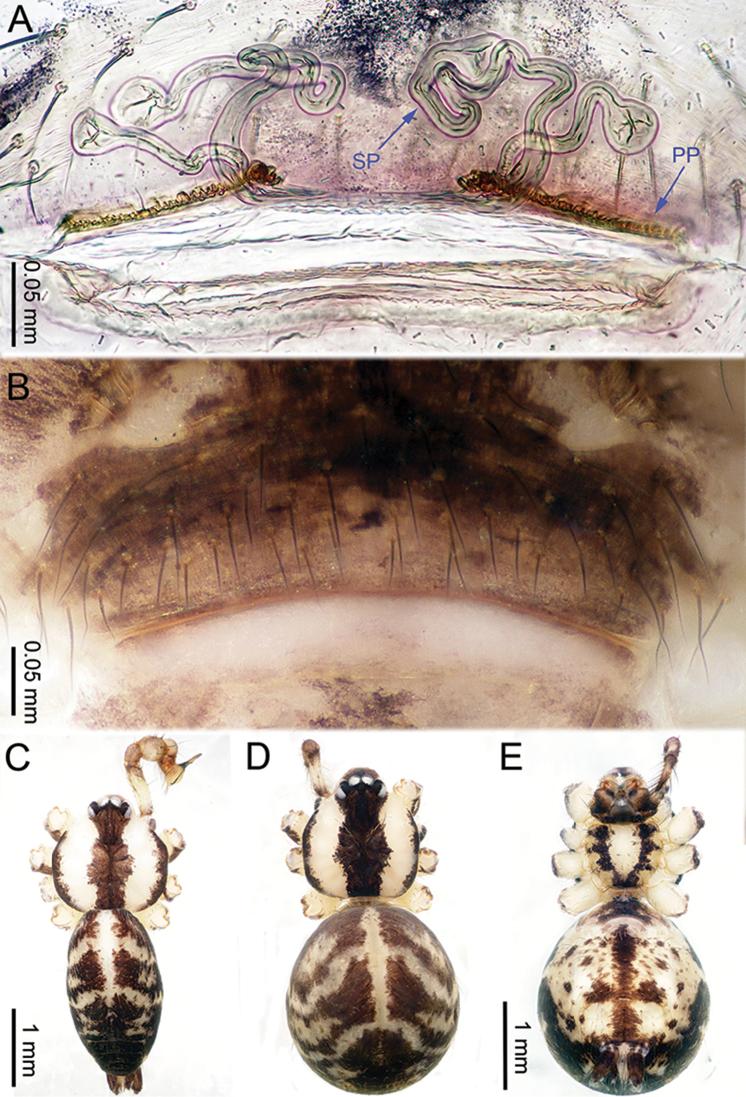
*Althepusxuae* Li & Li, sp. n., male holotype and female paratype. **A** Internal genitalia, dorsal view **B** Female epigastric furrow, ventral view **C** Male habitus, dorsal view **D** Female habitus, dorsal view **E** Female habitus, ventral view. Abbreviations: SP spermatheca; PP pore plate.

Figs 16 , 17 , 20 , 21

##### Types.

**Holotype**: ♂, China, Yunnan Province, Nujiang of the Lisu Autonomous Prefecture, Lushui County, Nouth of Pianma Town, 26°01.513'N, 98°37.313'E, 2125 m a.s.l., 27.VI.2016, M. Xu and Y. Li. **Paratypes**: 1♂2♀, same data as holotype.

##### Other material examined.

1♂, China, Yunnan Province, Nujiang of the Lisu Autonomous Prefecture, Lushui County, Pianma Town, Fengxue Yakou 25°59.628'N, 98° 39.697'E, 2337 m a.s.l., 29.VI.2016, M. Xu and Y. Li.

##### Etymology.

The specific epithet is a patronym in honour of Mingjie Xu who collected the types; noun (name) in genitive case.

##### Diagnosis.

*Althepusxuae* Li & Li, sp. n. resembles *A.chengmenensis* Li & Li, sp. n. in having a sigmoid conductor in the males, and curved, elongate spermathecae in the females. Males can be distinguished from the latter species by the smooth margin and blunt distal part of the conductor (Figure 16B); females can be distinguished by the two shorter spermathecae on each side (versus longer in *A.chengmenensis* Li & Li, sp. n.) (Figs 2A, 17A), can be distinguished from all the other known species of the genus by the lateral spermathecae having a longer stalk than medial spermathecae (Figure 17A).

##### Description.

**Male** (holotype). Total length 3.64; carapace 1.34 length, 1.40 width; abdomen 2.05 length, 1.24 width. Carapace round, yellow, with brown lateral margins and one wide, brown median band, the middle one wider than others. Anterior margin of cephalic region distinctly elevated (Figure 17C). Clypeus brown. Cheliceral promargin with two teeth, followed by a lamina, retromargin with two small teeth (Figure 20I), posterior surface of fang with 27 small denticles. Labium brown. Sternum yellow, with two longitudinal brown bands. Abdomen elongate, with complex patterns dorsally and ventrally (Figure 17C). Legs brown, femur and tibia with white annulations. Leg measurements: I missing, II 20.05 (5.32, 0.60, 5.13, 7.05, 1.75), III 12.82 (3.52, 0.59, 3.40, 4.00, 1.31), IV missing. Male palp (Figure 16A–D): tarsus with one retrolateral spine and one hooked spine with tip directed distally (Figure 16D); bristles at the top of cymbial protrusion (Figure 16A) as in *A.hongguangi* Li & Li, sp. n.; bulb yellow, ovate; embolus arising prolatero-proximally from bulb, slightly sigmoid; conductor arising retrolatero-distally from bulb, sigmoid; embolus and conductor widely separated (distance less than diameter of bulb).

**Female** (one of the paratypes). Total length 3.40; carapace 1.30 length, 1.20 width; abdomen 2.48 length, 1.85 width. Similar to male in colour and general features (Figure 17D–E), but smaller. Internal genitalia with two round spermathecae on long, slender stalks on each side and pores plate at the base (Figure 17A). Leg measurements: I missing, II missing, III 9.39 (2.64, 0.46, 2.40, 2.80, 1.09), IV 13.14 (3.80, 0.50, 3.52, 3.92, 1.40).

##### Variation.

Males: carapace 1.33–1.34 length, 1.40–1.44 width, leg I lost (the number of specimens = 2). Females: carapace 1.03–1.30 length, 1.20–1.25 width, leg I lost (the number of specimens = 2).

##### Distribution.

China. Yunnan Province (Figure 21).

##### Natural history.

Collected by sieving leaf litter in dark and moist environments.

#### Althepus yizhuang

Taxon classificationAnimaliaAraneaeOchyroceratidae

Li & Li sp. n.

http://zoobank.org/5647E1C8-8360-4333-A9A2-59806CACC6D0

**Figure 18.**
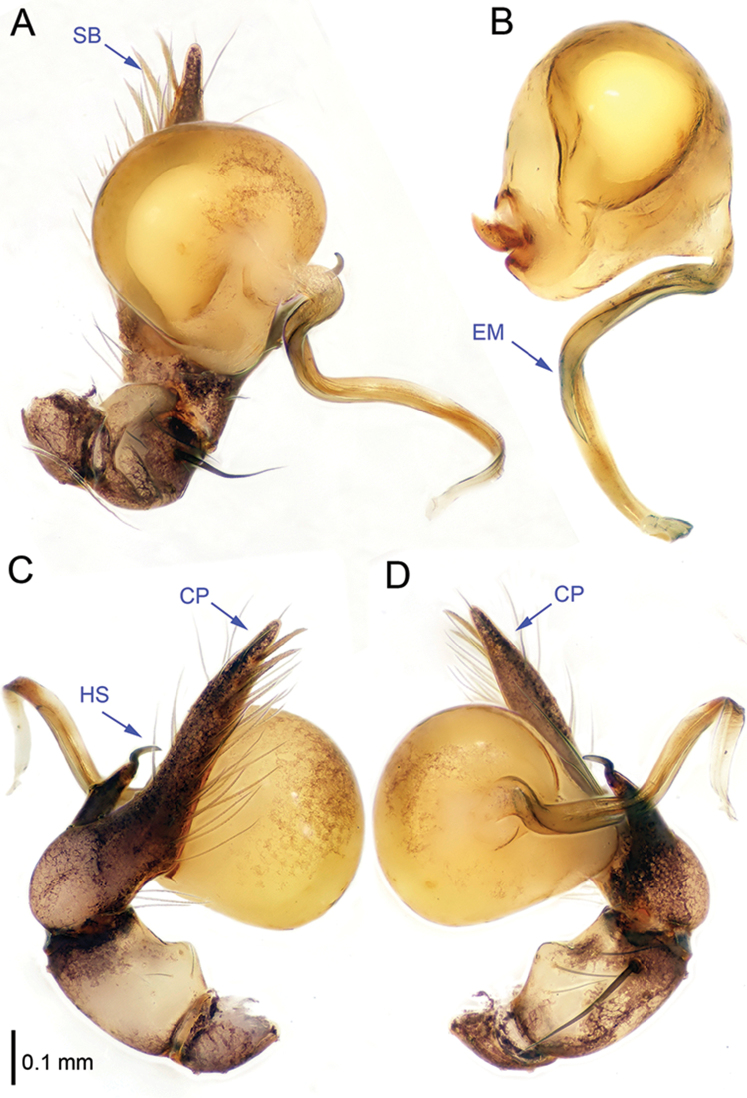
*Althepusyizhuang* Li & Li, sp. n., male holotype. **A** Palp, ventral view **B** Palpal bulb, prolateral view **C** Palp, prolateral view **D** Palp, retrolateral view. Abbreviations: CO conductor; EM embolus; CP cymbial protrusion; HS hook-shaped spine; SB serrated bristles.

**Figure 19.**
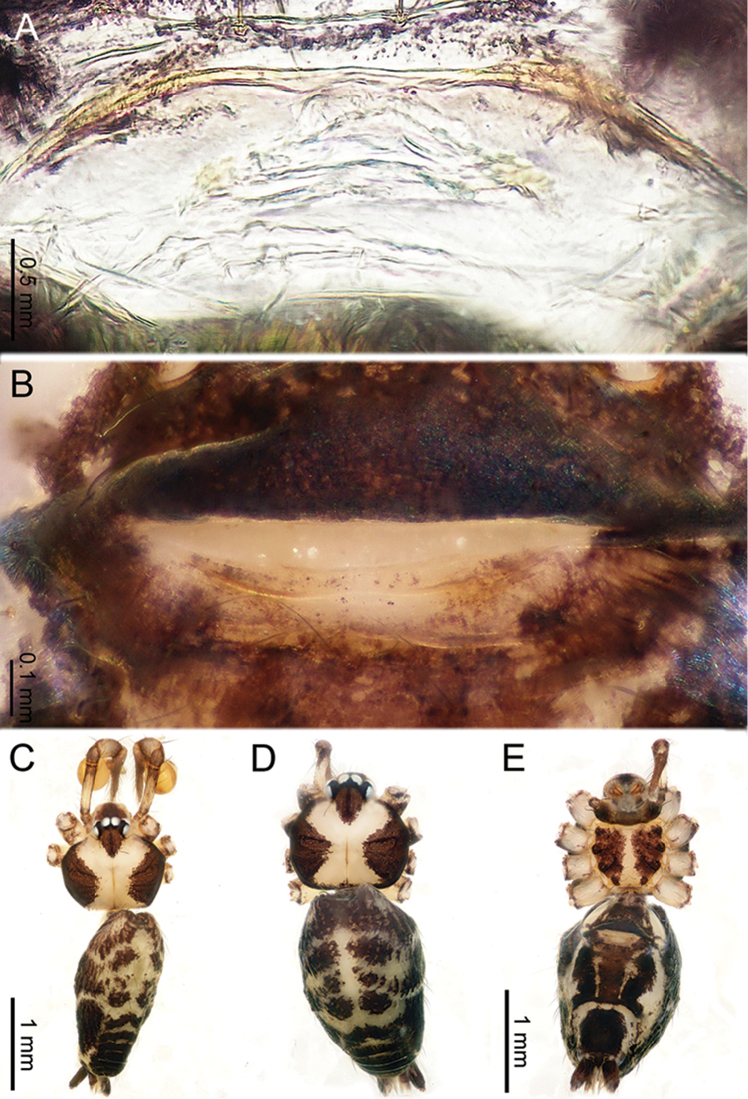
*Althepusyizhuang* Li & Li, sp. n., male holotype and female paratype. **A** Internal genitalia, dorsal view **B** Female epigastric furrow, ventral view **C** Male habitus, dorsal view **D** Female habitus, dorsal view **E** Female habitus, ventral view. Abbreviation: SP spermatheca.

**Figure 20.**
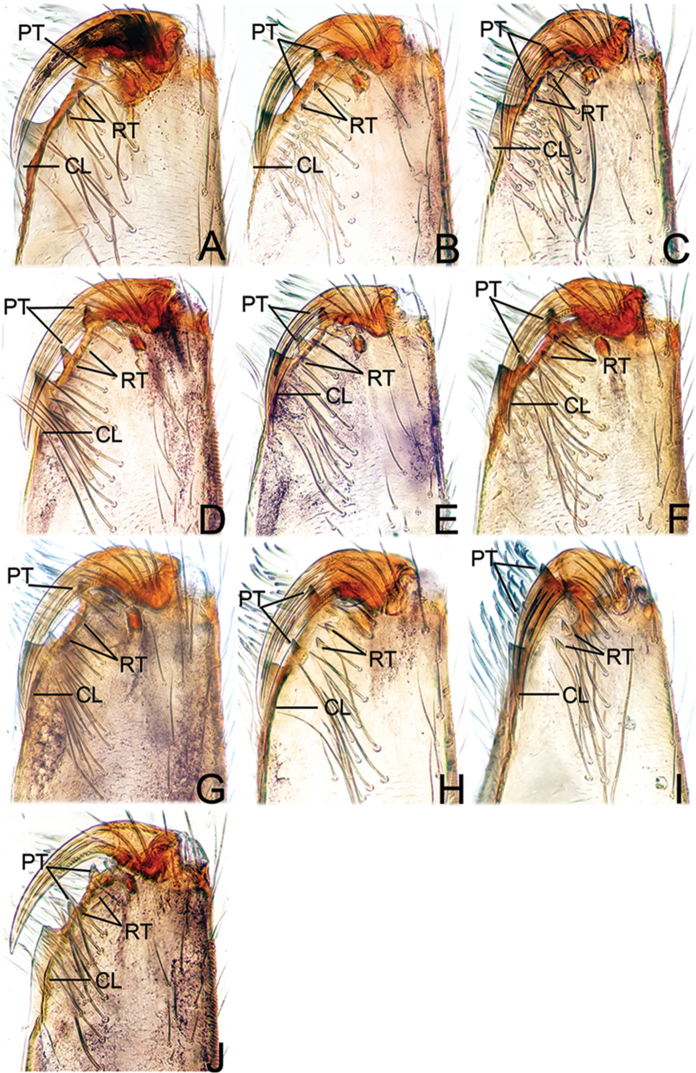
Cheliceral retromargin. **A***Althepuschengmenensis* Li & Li, sp. n. **B***A.cheni* Li & Li, sp. n. **C***A.gouci* Li & Li, sp. n. **D***A.hongguangi* Li & Li, sp. n., **E***A.phousalao* Li & Li, sp. n. **F***A.qianhuang* Li & Li, sp. n. **G***A.qingyuani* Li & Li, sp. n. **H***A.sepakuensis* Li & Li, sp. n. **I***A.xuae* Li & Li, sp. n. **J***A.yizhuang* Li & Li, sp. n. Abbreviations: PT promarginal teeth; RT retromarginal teeth; CL cheliceral lamina.

Figs 18 , 19 , 20 , 22

##### Types.

**Holotype**: ♂, Indonesia, Sumatra, West Sumatra Province, Sijunjung, Padang Sibusuk Village, Bukit Ponggang Cave, 00°44.245'S, 100°50.330'E, 278 m a.s.l., 27.V.2014, Z. Yao. **Paratypes**: 1♂2♀, same data as holotype.

##### Etymology.

The specific name is derived from the Chinese pinyin ‘yi zhuang’, which means ‘sigmoid’, referring to the sigmoid embolus (Figure 18); adjective.

##### Diagnosis.

*Althepusyizhuang* Li & Li, sp. n. can be distinguished from all other known species of the genus by the remarkably long and sigmoid embolus as well as by the absence of a conductor in males (Figure 18). Females are distinguished by inconspicuous spermathecae (Figure 19A).

##### Description.

**Male** (holotype). Total length 3.13; carapace 1.00 length, 1.14 width; abdomen 1.80 length, 0.94 width. Carapace round, yellow, with triangular brown margins and a narrow, brown median line behind ocular area (Figure 19C). Anterior margin of cephalic region distinctly elevated. Clypeus brown. Cheliceral promargin with two teeth, followed by a lamina, retromargin with two small teeth (Figure 20J), posterior surface of fang with 16 small denticles. Labium brown. Sternum brown, with a triangular yellow patch in the middle. Abdomen elongate, with complex patterns dorsally and ventrally (Figure 19C). Legs brown. Leg measurements: I 24.39 (5.83, 0.43, 6.22, 9.68, 2.23), II 14.81 (3.85, 0.41, 3.85, 5.45, 1.25), III 9.85 (2.81, 0.40, 2.50, 3.20, 0.94), IV 14.47 (4.17, 0.41, 3.91, 4.81, 1.17). Male palp (Figure 18A–D): tarsus with one hooked spine with tip directed proximally; cymbium slender (Figure 18C); bristles at the top of the cymbial protrusion (Figure 18A) as in *A.hongguangi* Li & Li, sp. n.; bulb bright yellow, ovate; embolus arising retrolatero-distally from bulb, bright yellow.

**Female** (one of the paratypes). Total length 3.13; carapace 0.85 length, 0.95 width; abdomen 1.90 length, 1.17 width. Similar to male in colour, general features and body size (Figure 19D–E). Internal genitalia with inconspicuous spermathecae (Figure 19A). Leg measurements: I missing, II 11.57 (3.00, 0.35, 2.97, 4.00, 1.25), III 8.21 (2.34, 0.36, 2.10, 2.53, 0.88), IV 11.52 (3.20, 0.38, 3.20, 3.60, 1.14).

##### Variation.

Males: carapace 1.00 length, 1.14–1.25 width; femur I 5.71–5.83 (holotype and paratypes with similar length).

##### Distribution.

Indonesia. Known only from the type locality (Figure 22).

##### Natural history.

Collected at a cave entrance at an altitude of 278 m.

##### Remark.

*Althepusyizhuang* Li & Li, sp. n., was labelled as “sp. 84” in the analysis of Li and Li (2018).

**Figure 21.**
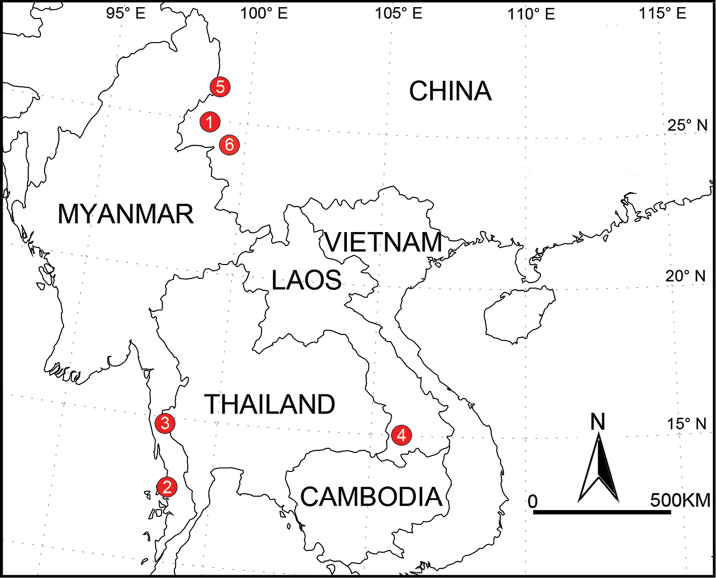
Known distribution of new *Althepus* species from Laos, Myanmar, and China. **1***A.chengmenensis* Li & Li, sp. n. **2***A.cheni* Li & Li, sp. n. **3***A.gouci* Li & Li, sp. n. **4***A.phousalao* Li & Li, sp. n. **5***A.xuae* Li & Li, sp. n. **6***A.qingyuani* Li & Li, sp. n..

**Figure 22.**
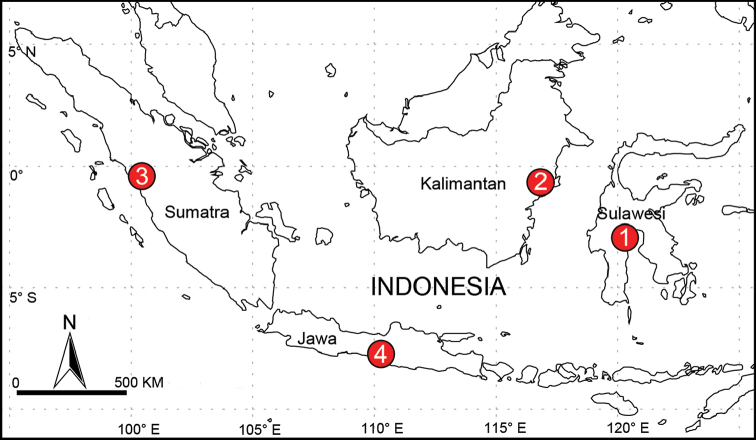
Known distribution of new *Althepus* species from Indonesia. **1***A.hongguangi* Li & Li, sp. n. **2***A.sepakuensis* Li & Li, sp. n. **3***A.yizhuang* Li & Li, sp. n. **4***A.qianhuang* Li & Li, sp. n..

## Discussion

In addition to morphological studies, we used molecular data from our extensive sampling to test the monophyly of the genus *Althepus* and delimitate the species (Li and Li 2018). The molecular topologies inferred by two different approaches all supported *Althepus* as a monophyletic group. The species delimitation inferred by three different approaches supported the evolutionary independence of 54 distinct lineages. For details, see *Althepus* sp. 23, *Althepus* sp. 84, *Althepus* sp. 97, *Althepus* sp. 119, and *Althepus* sp. 131 in figure 1 and supplementary figures S1–S4 of Li and Li (2018).

In this paper, we describe seven new species in lowland habitats of southern Indo-Burma, Sunda shelf islands, and three new species in highlands of northern Indo-Burma. The genus appears to have a higher diversity in lowlands compared to highlands. Recent studies indicate that this may be due to the repeated isolation and reconnection of Southeast Asian landmasses caused by sea-level fluctuations (Liu et al. 2017, Li and Li 2018).

## Supplementary Material

XML Treatment for Althepus

XML Treatment for Althepus chengmenensis

XML Treatment for Althepus cheni

XML Treatment for Althepus gouci

XML Treatment for Althepus hongguangi

XML Treatment for Althepus phousalao

XML Treatment for Althepus qianhuang

XML Treatment for Althepus qingyuani

XML Treatment for Althepus sepakuensis

XML Treatment for Althepus xuae

XML Treatment for Althepus yizhuang
